# Supplementary material for: Ultra-High-Performance Liquid Chromatography Quadrupole Time-of-Flight Mass Spectrometry for Simultaneous Pesticide Analysis and Method Validation in Sweet Pepper
Source: Molecules. 2023 Jul 22;28(14):5589. doi: 10.3390/molecules28145589 (PMC10383869; doi:10.3390/molecules28145589)
Supplement: Supplementary file 1 [file molecules-28-05589-s001.zip › molecules-2478248-Supplementary data.pdf]

Supplementary Material

Ultra-High-Performance Liquid Chromatography Quadrupole Time-of-Flight Mass Spectrometry for Simultaneous Pesticide Analysis and Method Validation in Sweet Pepper

Han Yeol Bang [1,†], Yong-Kyoung Kim [2,†], Hyoyoung Kim [2], Eun Joo Baek [2], Taewoong Na [2], Kyu Sang Sim [2] and Ho Jin Kim [2,*]

[1] Gyeongnam Provincial Office, National Agricultural Products Quality Management Service, Busan 47537, Republic of Korea; bbangkkc@korea.kr

[2] Experiment Research Institute, National Agricultural Products Quality Management Service, Gimcheon-si 39660, Republic of Korea; ykkim79@korea.kr (Y.-K.K.); hyo02@korea.kr (H.K.); qrgh1004@naver.com (E.J.B.); naratw@korea.kr (T.N.); sim9612@naver.com (K.S.S.)

* Correspondence: rex7878@korea.kr; Tel.: +82-54-429-7871

† These authors contributed equally to this work.

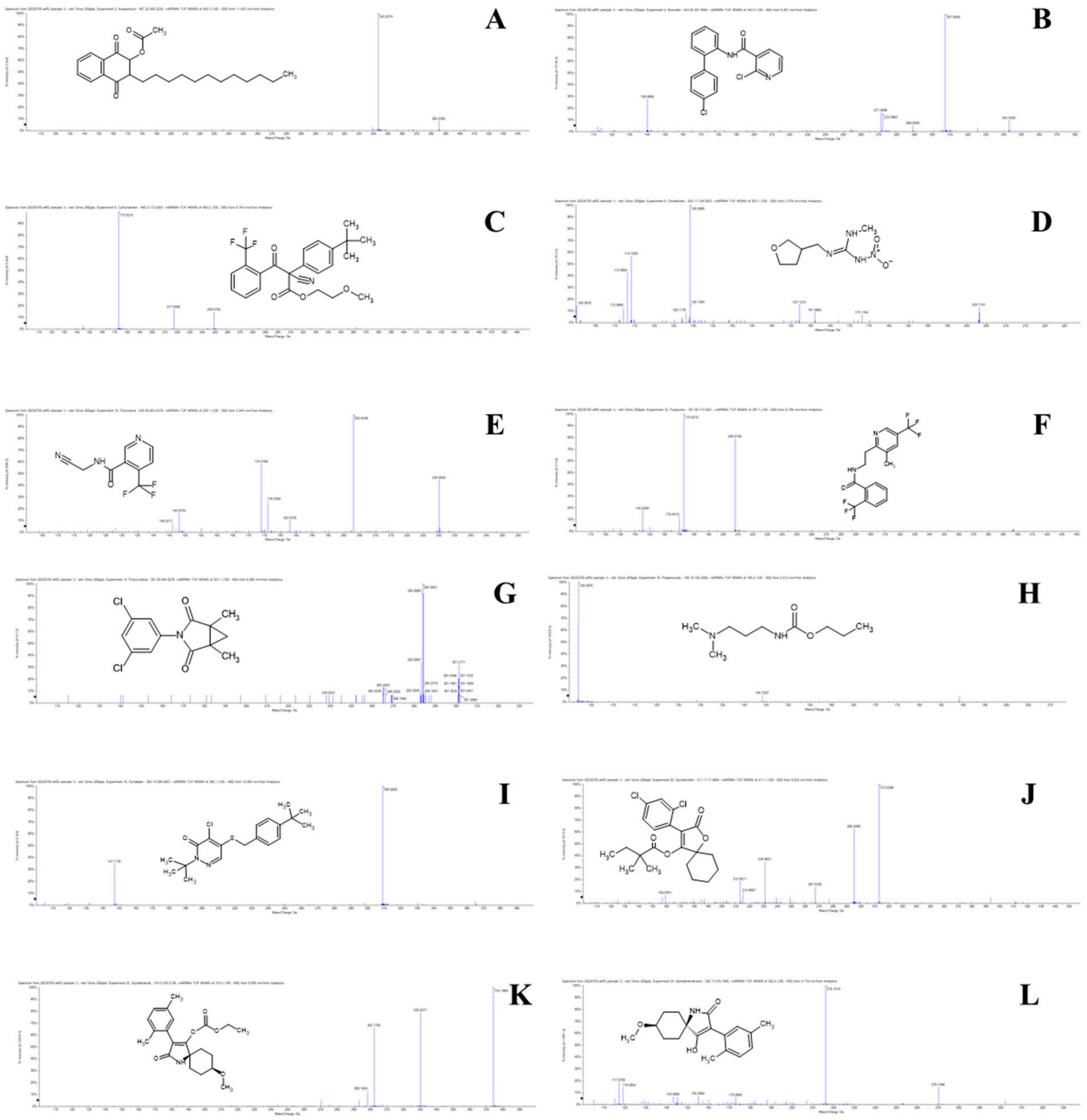

Supplementary Figure S1. Molecular structures and QTOF fragments of 12 pesticides. A) Acequinocyl, B) Boscalid, C) Cyflumetofen, D) Dinotefuran, E) Flonicamid, F) Fluopyram, G) Procymidone, H) Propamocarb, I) Pyridaben, J) Spirodiclofen, K) Spirotetramat, L) Spirotetramat-enol.

Supplementary Table S1. Parameters for the analysis of 140 pesticides by UHPLC-QTOF.

No.	Compound	Retention time (min)	Precursor ion (*m/z*)	Fragment ion (*m/z*)	Declustering potential (eV)	Collision energy (eV)
1	2,3,5-Trimethacarb	5.26	194.12	107.05	70	55
			194.12	137.10	70	10
2	3,4,5-trimethacarb	5.32	194.12	137.10	70	10
			194.12	122.07	70	35
3	6-Benzyl aminopurine	3.67	226.1	65.042	25	70
			226.1	91.05	25	35
4	Acequinocyl	11.66	407.26	343.23	30	13
			407.26	385.24	30	7
5	Acibenzolar-S-methyl	5.96	211	136.01	80	35
			211	68.98	80	75
6	Allethrin	9.15	303.2	123.12	105	20
			303.2	135.08	105	20
7	Ametoctradin	8.33	276.22	149.08	110	40
			276.22	176.09	110	45
8	Asulam	2.55	231.04	108.04	95	25
			231.04	92.05	95	25
9	Azamethiphos	3.92	324.98	183.00	70	20
			324.98	76.02	70	80
10	Benalaxyl	7.65	326.18	294.15	35	15
			326.18	91.05	35	45
11	Bensulide	7.34	398.07	218.03	105	21
			398.07	313.98	105	15
12	Boscalid	6.21	343.04	307.06	30	29
			343.04	271.09	30	43
13	Butocarboxim	3.55	213.07	156.04	95	15
			213.07	75.02	95	19
14	Carbetamide	3.84	237.12	118.09	80	15
			237.12	120.04	80	23
15	Chlorantraniliprole	5.55	483.98	285.92	40	17
			483.98	452.94	40	25
16	Chlorfenvinphos(*E*)	7.76	358.98	169.97	110	53
			358.98	98.98	110	33
17	Chlorfenvinphos(*Z*)	7.76	358.98	155.05	110	15
			358.98	204.94	110	27

18	Chlorfluazuron	9.97	539.97	346.96	120	53
			539.97	382.94	120	27
19	Chloridazone	3.22	222.04	65.04	20	49
			222.04	77.04	20	57
20	Chlorobenzuron	7.51	309.02	156.02	30	21
			309.02	75.02	30	89
21	Chlorotoluron	4.86	213.08	46.06	105	23
			213.08	72.04	105	39
22	Chloroxuron	6.72	291.09	46.06	130	45
			291.09	72.04	130	50
23	Clethodim sulfone	5.43	392.13	164.07	55	37
			392.13	208.14	55	27
24	Clethodim sulfoxide	5.52	376.13	164.07	95	23
			376.13	206.12	95	23
25	Clomeprop	8.97	324.06	120.08	115	31
			324.06	203.00	115	19
26	Crotoxyphos	6.23	332.1	211.03	46	15
			332.1	127.01	46	35
27	Crufomate	7.31	292.09	108.02	165	31
			292.09	236.03	165	27
28	Cyanazine	3.84	241	214.08	51	25
			241	104.00	51	43
29	Cyantraniliprole	4.52	475	285.92	30	21
			475	177.00	30	56
30	Cyazofamid	7.07	325	261.09	40	15
			325	108.01	40	22
31	Cyclaniliprole	7.12	599.92	177.00	40	22
			599.92	283.92	40	73
32	Cycloate	8.26	216.14	83.08	45	22
			216.14	63.02	45	45
33	Cycloprothrin	9.81	499.12	455.08	75	17
			499.12	257.02	75	20
34	Cyenopyrafen	9.81	394.25	254.13	30	39
			394.25	310.19	30	33
35	Cyflumetofen	8.71	465.2	173.02	75	25
			465.2	249.07	75	17
36	Demeton-S	5.73	259.06	61.01	30	55
			259.06	89.04	30	17

37	Demeton-S-methyl-sulfone	3.28	263	169.00	55	21
			263	109.00	55	31
38	Demeton-S-sulfone	3.27	291.05	234.99	115	21
			291.05	263.02	115	17
39	Demeton-S-sulfoxide	3.19	275.05	140.98	25	23
			275.05	80.97	25	55
40	Diclosulam	4.58	405.99	160.98	130	31
			405.99	377.97	130	21
41	Dimethoate	3.19	230.01	124.98	40	25
			230.01	170.97	40	19
42	Dinotefuran	2.59	203.11	129.09	125	15
			203.11	157.12	125	9
43	Disulfoton	8.08	275	89.04	51	25
			275	61.01	51	43
44	Disulfoton sulfone	4.94	307.03	124.98	105	23
			307.03	96.95	105	31
45	Disulfoton sulfoxide	4.82	291	185	39	19
			291	129	39	41
46	Dodine	8.15	228.24	43.05	45	37
			228.24	57.07	45	31
47	Emamectin B1a	9.36	886.53	158.11	150	53
			886.53	82.06	150	105
48	Fensulfothion	5.17	309.04	234.97	135	27
			309.04	252.98	135	23
49	Flonicamid	2.82	230.05	203.04	90	23
			230.05	148.04	90	37
50	Fluometuron	4.74	233.09	46.06	130	31
			233.09	72.04	130	39
51	Fluopyram	6.72	397.05	173.02	35	35
			397.05	208.01	35	27
52	Flupyradifurone	3.14	289.06	126.01	106	30
			289.06	72.98	106	95
53	Fluridone	5.65	330.11	259.10	150	63
			330.11	309.10	150	47
54	Fluthiacet-methyl	7.45	404.03	274.00	125	41
			404.03	344.01	125	33
55	Flutriafol	5.03	302.11	70.04	30	21
			302.11	75.02	30	93

56	Fluxametamide	9.55	474.06	160.04	20	51
			474.06	400.01	20	25
57	Imibenconazole	9.22	411	125.01	50	35
			411	89.04	50	111
58	Indaziflam	7.00	302.18	138.08	95	31
			302.18	145.10	95	29
59	Ipfencarbazone	7.57	427.05	198.07	120	17
			427.05	43.05	120	43
60	Isoproturon	5.16	207.15	165.10	115	19
			207.15	46.06	115	29
61	Isoxaben	6.32	333.18	107.01	120	89
			333.18	165.05	120	29
62	Isoxathion	7.91	314.06	105.03	115	21
			314.06	286.03	115	13
63	Lenacil	5.08	235.14	136.04	30	41
			235.14	153.07	30	19
64	Malaoxon	4.14	315.07	127.04	105	13
			315.07	99.01	105	35
65	Mandestrobin	7.66	314.18	119.05	55	35
			314.18	192.10	55	15
66	Mefentrifluconazole	7.87	398.09	43.03	35	147
			398.09	70.04	35	39
67	Mephosfolan	3.98	270.04	139.96	25	35
			270.04	196.02	25	17
68	Metaflumizone(*E*)	9.10	507.12	178.04	70	35
			507.12	287.08	70	35
69	Metamitron	3.16	203.09	104.05	100	29
			203.09	175.10	100	21
70	Metominostrobin(*E*)	5.41	285.12	194.06	30	25
			285.12	196.08	30	21
71	Neburon	7.35	275.07	57.07	120	27
			275.07	88.11	120	21
72	Nitenpyram	2.69	271.1	126.01	35	35
			271.1	56.05	35	35
73	Norea	6.05	223.18	135.12	100	25
			223.18	67.05	100	39
74	Norflurazon	5.33	304.05	160.04	135	37
			304.05	284.04	135	31

75	Orthosulfamuron	5.10	425.12	199.08	120	15
			425.12	227.05	120	21
76	Orysastrobin	6.40	392.19	116.05	55	33
			392.19	205.10	55	17
77	Oxadiargyl	7.92	341.05	151.02	71	33
			341.05	222.00	71	31
78	Oxamyl oxime	2.78	163.05	44.98	55	47
			163.05	46.99	55	41
79	Oxathiapiprolin	6.35	540.15	500.14	195	37
			540.15	522.14	195	33
80	Oxycarboxin	3.32	268.06	175.00	40	23
			268.06	43.01	40	55
81	Oxydemeton-methyl	2.72	247.02	105.04	35	15
			247.02	109.00	35	33
82	Pebulate	8.15	204.14	128.11	55	15
			204.14	41.04	55	43
83	Phenmedipham	5.56	301.12	136.04	120	31
			301.12	65.04	120	73
84	Phenothrin	10.52	351.2	249.13	110	17
			351.2	305.19	110	15
85	Phorate	7.93	261.02	46.99	30	43
			261.02	75.02	30	15
86	Phorate oxon	5.47	245.04	46.99	55	43
			245.04	75.02	55	23
87	Phorate oxon sulfone	3.28	277.03	183.03	115	15
			277.03	80.97	115	77
88	Phorate oxon sulfoxide	3.14	261.04	110.97	90	27
			261.04	243.03	90	11
89	Phorate sulfone	4.98	293.01	247.03	110	9
			293.01	96.95	110	49
90	Phorate sulfoxide	4.80	277.02	142.94	90	27
			277.02	170.97	90	17
91	Phosfolan	3.51	256.02	139.96	80	29
			256.02	227.99	80	19
92	Picarbutrazox	7.21	410.19	107.06	80	37
			410.19	310.14	80	19
93	Picolinafen	9.05	377.09	238.05	55	31
			377.09	359.09	55	27

94	Procymidone	6.85	301.05	284.02	30	10
			303.05	286.02	25	15
95	Propamocarb	2.55	189.16	102.05	30	25
			189.16	74.02	30	33
96	Propyrisulfuron	6.41	456.09	196.07	145	21
			456.09	261.03	145	21
97	Proquinazid	9.90	373.04	271.92	25	45
			373.04	288.95	25	31
98	Prosulfocarb	8.60	252.14	65.04	85	79
			252.14	91.05	85	39
99	Pydiflumetofen	8.25	426.03	192.94	160	49
			426.03	406.03	160	19
100	Pyflubumide	9.32	536.23	111.09	160	79
			536.23	155.08	160	33
101	Pyflubumide-NH	8.92	466.19	137.07	205	51
			466.19	382.18	205	45
102	Pyraclonil	4.77	315.11	241.12	125	29
			315.11	276.09	125	21
103	Pyraclostrobin	7.87	388.11	104.05	40	75
			388.11	163.06	40	37
104	Pyraziflumid	6.82	380.08	147.02	150	41
			380.08	362.08	150	21
105	Pyrazosulfuron-ethyl	6.67	415.1	182.06	25	31
			415.1	83.02	25	67
106	Pyrazoxyfen	7.62	403.06	65.04	95	109
			403.06	91.05	95	51
107	Pyridaben	10.07	365.14	309.08	90	19
			365.14	147.12	90	35
108	Pyriofenone	8.11	366.11	184.01	55	29
			366.11	209.08	55	35
109	Secbumeton	4.94	226.17	100.05	75	39
			226.17	170.10	75	23
110	Sedaxane	6.64	332.16	159.03	80	23
			332.16	292.14	80	21
111	Sethoxydim	8.88	328.19	178.09	75	27
			328.19	282.15	75	17
112	Simazine	4.15	202.09	132.03	110	23
			202.09	68.02	110	45

113	Spinosyn A	8.07	732.47	142.12	35	51
			732.47	98.09	35	91
114	Spinosyn D	8.56	746.48	142.12	45	39
			746.48	98.09	45	69
115	Spirodiclofen	9.81	411.11	313.03	115	31
			411.11	230.96	115	15
116	Spirotetramat	6.76	374.2	330.21	40	17
			374.2	302.18	40	23
117	Spirotetramat-enol	4.68	302.17	216.10	125	37
			302.17	270.15	125	27
118	Sulfentrazone	4.33	404.02	307.00	50	35
			404.02	386.99	50	21
119	Sulprofos	9.46	323.04	218.9749	100	21
			323.04	247.0066	100	15
120	Tebufloquin	7.52	290.16	192.08	35	43
			290.16	248.15	35	23
121	Tebufloquin M1	5.86	248.14	192.08	160	33
			248.14	232.11	160	49
122	Tebuthiuron	4.28	229.11	116.03	30	35
			229.11	172.09	30	23
123	Tepraloxydim	6.71	342.15	166.09	55	25
			342.15	250.15	55	17
124	Terbufos	8.93	289.05	103.06	35	11
			289.05	57.07	35	29
125	Terbufos oxon	6.78	273.07	103.06	80	13
			273.07	57.07	80	31
126	Terbufos oxon sulfone	3.75	305.06	202.96	115	23
			305.06	249.00	115	13
127	Terbufos oxon sulfoxide	3.57	289.07	142.99	80	27
			289.07	171.02	80	17
128	Terbufos sulfone	5.85	321.04	114.96	110	27
			321.04	96.95	110	53
129	Terbufos sulfoxide	5.85	305.05	187.00	25	13
			305.05	96.95	25	43
130	Tolfenpyrad	9.11	384.15	154.08	30	59
			384.15	197.10	30	35
131	Triafamone	4.22	407.06	160.09	150	57
			407.06	245.11	150	43

132	Triazamate	6.77	315.15	226.10	60	15
			315.15	72.04	60	43
133	Tribufos	10.10	315.1	168.99	100	21
			315.1	57.07	100	31
134	Tricyclazole	3.46	190.04	136.02	115	37
			190.04	65.04	115	63
135	Trifloxysulfuron	4.98	438.07	182.06	120	23
			438.07	83.02	120	63
136	Triticonazole	6.92	318.14	43.03	45	99
			318.14	70.04	45	27
137	TZ-1E	7.06	410.19	107.06	40	35
			410.19	310.14	40	19
138	Valifenalate	6.63	399.17	116.07	65	25
			399.17	155.03	65	37
139	Vernolate	8.16	204.14	128.11	51	15
			204.14	43.05	51	21
140	XMC	4.72	180.1	123.08	100	21
			180.1	51.02	100	79
